# New Books

**Published:** 2007-04

Analysis of Global Change Assessments: Lessons Learned

Committee on Analysis of Global Change Assessments, National Research Council

Washington, DC:National Academies Press, 2007. 206 pp. ISBN: 0-309-10544-7, $38.48

Assessments of Regional and Global Environmental Risks: Designing Processes for the Effective Use of Science in Decisionmaking

Alexander E. Farrell, Jill Jäger, eds.

Washington, DC:RFF Press, 2006. 293 pp. ISBN: 1-933115-04-1, $70

Creating a Climate for Change

Susanne C. Moser, Lisa Dilling, eds.

Cambridge, UK:Cambridge University Press, 2007. 576 pp. ISBN: 0-521-86923-2, $146

Emissions Trading: Principles and Practice, 2nd ed.

T.H. Tietenberg

Washington, DC:RFF Press, 2006. 223 pp. ISBN: 1-933115-30-0, $80

Environmental Disasters, Natural Recovery and Human Responses

Roger del Moral, Lawrence R. Walker

Cambridge, UK:Cambridge University Press, 2007. 216 pp. ISBN: 0-521-86034-5, $127

Environmental Protection, Law and Policy, 2nd ed.

Jane Holder, Maria Lee

Cambridge, UK:Cambridge University Press, 2007. 800 pp. ISBN: 0-521-69026-3, $58

Extreme Conflict and Tropical Forests

Wil De Jong, Deanna Donovan, Kenichi Abe, eds.

New York:Springer, 2007. 184 pp. ISBN: 1-4020-5461-7, $99

Fate of Persistent Organic Pollutants in the North Sea

Tatjana P. Ilyina

New York:Springer, 2007. 132 pp. ISBN: 3-540-68162-5, $89.95

From the Corn Belt to the Gulf: Societal and Environmental Implications of Alternative Agricultural Futures

Joan Iverson Nassauer, Mary V. Santelmann, Donald Scavia, eds.

Washington, DC:RFF Press, 2007. 260 pp. ISBN: 1-933115-47-4, $85

Gaia’s Revenge: Climate Change and Humanity’s Loss

P.H. Liotta, Allan W. Shearer

Westport, CT:Greenwood, 2007. 208 pp. ISBN: 0-275-98797-3, $49.95

Global Environmental Health in the 21st Century: From Governmental Regulation to Corporate Social Responsibility

Myron Harrison, Christine Coussens

Washington, DC:National Academies Press, 2007. 126 pp. ISBN: 0-309-10380-0, $26.10

Human Impacts on Weather and Climate, 2nd ed.

William R. Cotton, Roger A. Pielke Sr.

Cambridge, UK:Cambridge University Press, 2007. 330 pp. ISBN: 0-521-84086-6, $127

Introduction to Chemical Transport in the Environment

John C. Gulliver

Cambridge, UK:Cambridge University Press, 2007. 304 pp. ISBN: 0-521-85850-2, $98

Nanoparticles and Occupational Health

Andrew D. Maynard, David Y.H. Pui, eds.

New York:Springer, 2007. 186 pp. ISBN: 978-1-4020-5858-5,$109

Personal Care Compounds in the Environment: Pathways, Fate and Methods for Determination

Kai Bester

Hoboken, NJ:John Wiley & Sons, Inc., 2007. 263 pp. ISBN: 3-527-31567-3, $165

Progress in Preventing Childhood Obesity: How do We Measure Up?

Committee on Progress in Preventing Childhood Obesity

Washington, DC:National Academies Press, 2007. 494 pp. ISBN: 0-309-10208-1, $46.50

Quantified Eco-Efficiency

Gjalt Huppes, Masanobu Ishikawa, eds.

New York:Springer, 2007. 330 pp. ISBN: 1-4020-5398-6, $169

Reality Check: The Nature and Performance of Voluntary Environmental Programs in the United States, Europe, and Japan

Richard D. Morgenstern, William A. Pizer, eds.

Washington, DC:RFF Press, 2007. 185 pp. ISBN: 1-933115-36-8, $80

Scientific Writing: A Reader and Writer’s Guide

Jean-Luc Lebrun

Hackensack, NJ:World Scientific Publishing Co., 2007. 150 pp. ISBN: 981-270-473-6, $55

The Economics of Climate Change

Nicholas Stern

Cambridge, UK:Cambridge University Press, 2007. 712 pp. ISBN: 0-521-70080-1, $58

